# Sustainable synthesis of zinc oxide nanoparticles from *Persicaria lapathifolia*: versatile anticancer and antibacterial applications

**DOI:** 10.3389/fmolb.2025.1601811

**Published:** 2025-07-07

**Authors:** Essam Nageh Sholkamy, Mohamed A. A. Abdelhamid, Hadjer Rebai, Ananth Sivapunniyam, Mi-Ran Ki, Seung Pil Pack, Hazim O. Khalifa

**Affiliations:** ^1^ Department of Botany and Microbiology, College of Science, King Saud University, Riyadh, Saudi Arabia; ^2^ Biology Department, Faculty of Education and Arts, Sohar University, Sohar, Oman; ^3^ Department of Botany and Microbiology, Faculty of Science, Minia University, Minia, Egypt; ^4^ Laboratory of Molecular and Cellular Biology, Constantine 1- Frères Mentouri University, Chaâbat Erssas Campus, Constantine, Algeria; ^5^ Center for Applied Research, Saveetha School of Engineering, Saveetha Institute of Medical and Technical Sciences (SIMATS), Chennai, Tamil Nadu, India; ^6^ Department of Biotechnology and Bioinformatics, Korea University, Sejong, Republic of Korea; ^7^ Institute of Industrial Technology, Korea University, Sejong, Republic of Korea; ^8^ Department of Veterinary Medicine, College of Agriculture and Veterinary Medicine, United Arab Emirates University, Al Ain, United Arab Emirates; ^9^ UAEU Center for Public Policy and Leadership, United Arab Emirates University, Al Ain, United Arab Emirates

**Keywords:** colorectal cancer, zinc oxide nanoparticles, green synthesis, Persicaria lapathifolia, apoptosis, ROS, antibacterial activity

## Abstract

Colorectal cancer (CRC) is one of the most common cancers in the world and one of the leading causes of cancer mortality. In this study, zinc oxide nanoparticles (PlS-ZnO NPs) were synthesized eco-friendly using an aqueous extract from the stems of *Persicaria lapathifolia* and their anticancer and antibacterial activities were evaluated. The PlS-ZnO NPs were prepared by a simple sol-gel combustion method and investigated by different spectroscopic and microscopic techniques. The resulting nanoparticles were of polygonal and hexagonal morphology with the average size of 21.45 nm. The PlS-ZnO NPs exhibited high cytotoxicity against human colorectal cancer cell line (HCT-116) with IC50 value of 11.31 μg/mL. Cytomorphological studies showed that these nanoparticles killed cells through both apoptosis and necrosis. Apoptotic process was accompanied by enhancement of intracellular ROS and MMP. Moreover, the PlS-ZnO NPs exhibited excellent antibacterial activity towards different pathogenic bacteria like *Bacillus subtilis*, *Bacillus megaterium*, *Vibrio cholerae* and *Proteus vulgaris* and was found to be better than the positive control amoxicillin. These results indicate that the green synthesized PlS-ZnO NPs can be considered as a potential multifunctional agent for the treatment of colorectal cancer and bacterial infections. The environmentally friendly and cost-effective synthesis of PlS-ZnO nanoparticles (NPs) utilizing *P. lapathifolia* enhances the potential of this nanomaterial for medical applications. This sustainable approach not only reduces environmental impact but also aligns with the increasing demand for biocompatible materials in healthcare.

## 1 Introduction

Colorectal cancer (CRC) is a significant public health challenge, consistently identified as one of the foremost contributors to cancer mortality across the globe. It ranks as the third most frequently diagnosed cancer in men and holds the position of the second most common cancer among women (Bray et al., 2024). Globally, it is estimated that CRC contributed to 608,000 deaths, accounting for 8% of all cancer-related deaths. Furthermore, projections indicate that approximately 2.5 million individuals worldwide will develop CRC (Bray et al., 2024; Dizon and Kamal, 2024). Conventional cancer treatment approaches, such as adjuvant therapies (chemotherapy or radiotherapy) followed by surgical intervention, often result in adverse effects. Additionally, the untargeted distribution of traditional drugs has exacerbated the issue of drug resistance. This has prompted the scientific community to intensify the search for novel, environmentally friendly, and side-effect-free alternative therapies (Abd El-Hafeez et al., 2018). Apoptosis, the well-established process by which anticancer agents eliminate irreparably damaged cells, is a critical mechanism governing the normal cell transformation into a tumor cell and its subsequent metastasis, which is driven by mutations that dysregulate or circumvent the apoptotic pathway (Morgan et al., 2023; Wolf et al., 2023).

Bacterial infections represent a major global health concern, further exacerbated by the rise of antibiotic-resistant strains (Ahmed et al., 2015; Khalifa et al., 2024).

The biosynthesis of nanoparticles through eco-friendly and sustainable methods has gained considerable interest in various scientific fields, particularly in medicine and nanotechnology. Green synthesis techniques present several advantages over traditional chemical methods, including lower environmental impact, improved cost-effectiveness, and the utilization of natural resources (Ashraf et al., 2023; Sholkamy et al., 2024). In this context, the biosynthesis of nanoparticles (NPs) using plant extracts offers remarkable physicochemical properties and enhanced biological activities, which have generated significant interest in their application for cancer therapies (Ifeanyichukwu et al., 2020; Tang and Lv, 2014). The unique physicochemical properties of ZnO nanoparticles, including their small size, large surface area, and inherent antimicrobial and anticancer activities, make them an attractive option for addressing these challenges (Khezerlou et al., 2018; Moritz and Geszke-Moritz, 2013). Their wide band gap and high excitation binding energy enable them to effectively absorb ultraviolet light while remaining transparent to visible light. These distinctive properties set ZnO NPs apart from their bulk counterparts, facilitating their use in a range of applications (Kulkarni and Shirsat, 2015). Recent research has highlighted the promising anticancer (Karimzadeh et al., 2020; Pei et al., 2023) and antibacterial (Kebede Urge et al., 2023; Mallikarjunaswamy et al., 2020; Shaban et al., 2022) properties of phyto-mediated ZnO NPs. The variability in biological activities is often attributed to the diverse compositions of the phytochemicals involved in the synthesis process. These phytochemicals play a crucial role in capping and stabilizing the nanoparticles, thereby enhancing their bioavailability (Hayat et al., 2024).

The toxicity of nanoparticles is closely linked to their physical characteristics and their ability to generate reactive oxygen species (ROS). These ROS can induce apoptosis in cancer cells and exhibit bactericidal effects (Mushtaq et al., 2023). Notably, ZnO NPs exhibit selective cytotoxicity towards cancer cells while sparing normal cells, making them a desirable candidate for anticancer agents (Li et al., 2023). Consequently, ZnO NPs are being explored as potential alternatives to synthetic drugs in both anticancer and antimicrobial applications, owing to their high bioavailability and efficacy.

Recent research has highlighted the synthesis of various nanoparticles, including iron oxide (Şahin et al., 2024), zero-valent selenium oxide (Johnson et al., 2024), and zinc oxide (Bouttier-Figueroa et al., 2024; Nazneen and Sultana, 2024), utilizing plant stem extracts. These nanoparticles exhibit remarkable antibacterial, antioxidant, anticancer, and photocatalytic properties. Notably, the synthesis of ZnO nanoparticles from *Persicaria bistoria* has been documented, showcasing antioxidant capabilities (Safavi et al., 2019). However, this remains the only study focused on nanoparticle synthesis within the *Persicaria* genus. Numerous studies have demonstrated the efficacy of various plant extracts in the green synthesis of zinc oxide (ZnO) nanoparticles, including *Ocimum sanctum* (Varghese et al., 2024), *Aloe vera* (Chaudhary et al., 2019), and *Citrus limon* (Sakthivel et al., 2022). These investigations underscore the potential of natural sources to facilitate environmentally friendly synthesis methods. *P. lapathifolia*, commonly known as the knotweed, curly top, or smartweed, is an ethnobotanically significant plant belonging to the Polygonaceae family. The diverse family of plants under consideration includes over 1,100 edible flowering species distributed across 46 genera (Ashraf et al., 2023; Sahidin et al., 2020). Notably, the genus *Persicaria* comprises around 150 species, predominantly found in tropical and subtropical regions worldwide ((Ashraf et al., 2023). Among these, *Persicaria lapathifolia* stands out as a robust herbaceous plant, reaching heights of up to 80 cm, and is easily recognizable by its striking red stem and alternately arranged leaves. This species thrives in moist soils and agricultural environments, making it a common presence across various ecological conditions.

Traditionally, *P. lapathifolia* has been utilized in folk medicine for its therapeutic benefits, with applications ranging from treating fever and dysentery to managing burns (Al-Sodany et al., 2024). Recent investigations have begun to reveal the extensive pharmacological potential of this plant, emphasizing its antimicrobial, anticancer, anti-inflammatory, antioxidant, and enzyme inhibitory activities, such as anticholinesterase and glycosidase inhibition. These bioactive properties are largely attributed to the diverse array of secondary metabolites found within the plant, including flavonoids, acylated flavonoids, chalcones, ferulate esters, and phenolic compounds (Gangwar et al., 2024; Hailemariam et al., 2018; Idoudi et al., 2024; Rekha et al., 2019).

Despite its recognized ethnobotanical and pharmacological significance, the potential of *P. lapathifolia* for the biosynthesis of ZnO NPs has yet to be explored, presenting an exciting avenue for future research. We aim to synthesize and characterize ZnO NPs using the aqueous extract from the stems of *P. lapathifolia*. Furthermore, we will evaluate the *in vitro* antibacterial activity and antiproliferative effects of these synthesized nanoparticles against the HCT-116 colorectal cancer cell line. This research not only contributes to the understanding of *P. lapathifolia*’s potential in nanomedicine but also promotes eco-friendly synthesis methods for developing multifunctional nanomaterials.

## 2 Materials and methods

### 2.1 Chemical reagents

A high-purity analytical-grade reagents were utilized for the synthesis and characterization of ZnO NPs. To ensure optimal cell culture conditions, Dulbecco’s Modified Eagle Medium (DMEM) and fetal bovine serum (FBS) were sourced from Thermo Fisher Scientific (United States). Cytotoxicity assays were performed using 3-(4,5-dimethylthiazol-2-yl)-2,5-diphenyl tetrazolium bromide (MTT), along with essential antibiotics such as penicillin and streptomycin, sourced from Himedia Laboratories Pvt. Ltd. Zinc nitrate hexahydrate was used as the precursor for nanoparticle synthesis, and dimethyl sulfoxide (DMSO) served as the solvent. For the assessment of cell viability and apoptosis, a variety of fluorescent dyes were utilized, including ethidium bromide, dichlorodihydrofluorescein diacetate (DCFH-DA), acridine orange, propidium iodide, and rhodamine 123, all procured from Sigma-Aldrich (United States). These reagents were carefully selected for their reliability and effectiveness in the assays conducted throughout the investigation.

### 2.2 Phytochemical screening

Fresh and healthy *P. lapathifolia* plants were purchased from State Horticulture Farm, Aduthurai, TamilNadu, India (Latitude: 11° 00′55.51″N; Longitude: 79° 28′51.35″E). The stem extract of *P. lapathifolia* was prepared using water, methanol, and chloroform as solvents. Specifically, 50 gm of stem powder was dissolved in 1000 mL of each solvent and extracted using a Soxhlet apparatus at a temperature of 60°C. Preliminary phytochemical screening was conducted following the methodologies outlined by Harborne (Harborne, 1998) and Trease & Evans (Trease and Evans, 1983), which allowed for the identification of the various phytochemical constituents present in the extract.

### 2.3 Carcinoma cell culture

The HCT-116 colorectal carcinoma cell line and the normal human colon cell line (CCD 841 CoN) were acquired from the National Centre for Cell Sciences in Pune, India. Both cell lines were cultured in DMEM supplemented with 10% FBS and antibiotics, specifically streptomycin (100 mg/L) and penicillin (100 U/mL), to inhibit bacterial contamination. Cells were maintained as monolayers in a humidified incubator at 37°C with a 5% CO_2_ atmosphere to support optimal growth and proliferation. The culture conditions were closely monitored, and cells were passaged when they reached approximately 80% confluence to ensure a steady supply for experimental procedures.

### 2.4 Biosynthesis of PlS-ZnO NPs

To prepare the plant material for nanoparticle synthesis, the stems of *P. lapathifolia* were thoroughly washed with tap water, followed by three rinses with distilled water to eliminate any surface contaminants. The cleaned stems were then chopped into small pieces and air-dried in the shade for 10 days. Once dried, the stems were ground into a fine powder using a mechanical blender. For the biosynthesis of PlS-ZnO NPs, 5 g of the powdered stem material was combined with 100 mL of deionized water and heated to 80°C for 30 min to produce a stem decoction. After cooling to room temperature, the mixture was filtered through muslin cloth, and the resulting filtrate was further refined using Whatman No. 1 filter paper to ensure a clean filtrate devoid of impurities. In parallel, a 0.1 M solution of zinc nitrate hexahydrate was prepared by dissolving 2.9748 g of the salt in 100 mL of deionized water. Subsequently, 10 mL of the filtered stem extract was added to 50 mL of the zinc nitrate solution. The reaction mixture was then maintained at 80°C for 2 h, during which a yellow gel-like consistency formed, indicating the formation of zinc oxide nanoparticles. The semi-solid product was subjected to calcination at 600°C for 2 h to enhance crystallinity and facilitate the conversion of the gel into solid nanoparticles (Gangwar et al., 2024). The resultant calcined powder was ground into a fine powder for further characterization and analysis.

### 2.5 Characterization of PlS-ZnO NPs

ZnO-NPs’ optical properties were evaluated using UV-visible spectroscopy at variable wavelengths ranging between 200 and 800 nM. The synthesized PlS-ZnO NPs’ band gap energy was determined utilizing formula E = hc/λ. The functional groups in PlS-ZnO NPs were determined with FTIR analysis in wavelength extending between 4000 and 400 cm^−1^. The crystallographic structure and average crystallite size were analyzed via X-ray diffraction (XRD) using Cu-Kα radiation (λ = 1.5406 Å), with 2θ scanning angles between 20° and 80°. The average crystallite size was estimated using the Debye–Scherrer equation. Surface morphology and elemental composition were investigated through Scanning Electron Microscopy (SEM) coupled with Energy-Dispersive X-ray Spectroscopy (EDX) using a ZEISS instrument (Germany).

### 2.6 Determination of ZnO-NPs’ cytotoxicity potential

The cytotoxic potential of ZnO NPs was evaluated using a rapid calorimetric assay adapted from the methodology previously described (Mosmann, 1983). HCT-116 colorectal carcinoma cells were employed for this assessment. A total of 1 × 10^4^ viable cells/mL were seeded into a 96-well tissue culture-treated plate, where they were exposed to varying concentrations of ZnO NPs (ranging from 2.5 to 15 μg/mL) and cultured for 24 h. A control group was maintained without the addition of PlS-ZnO NPs to establish baseline cell viability. Following the incubation period, the culture medium was replaced with an MTT solution (5 mg/mL) and the cells were incubated for an additional 4 h. This process facilitated the formation of a purple formazan precipitate, which indicates the presence of viable cells. The precipitate was then dissolved in 100 µL of DMSO, and absorbance was measured at 540 nm using a multi-plate reader to evaluate cell viability. Cell viability percentages were calculated by comparing the absorbance values of the control and experimental samples. The half-maximal inhibitory concentration (IC_50_) was determined from the dose-response curve derived from the data. To further assess the specificity of the ZnO NPs, the experiment was also conducted on normal human colon cells (CCD 841 CoN), enabling a comparative analysis of cytotoxic effects. This entire experimental procedure was performed in triplicate to ensure the reproducibility and reliability of the results.

#### 2.6.1 Intracellular ROS measurement

The generation of intracellular ROS was evaluated using the DCFH-DA staining assay, following the methodology described by Pereira et al. (Pereira et al., 1999). For this experiment, 2 × 10^6^ HCT-116 cells/mL were seeded into 6-well plates and incubated for 24 h to allow adhesion prior to treatment with various concentrations of PlS-ZnO NPs. An unexposed control group was also maintained for comparative purposes. After an overnight incubation, the cells were transferred to a 24-well plate and treated with selected concentrations of PlS-ZnO NPs (10 and 12.5 μg/mL). Following a 24-h treatment period, the cells were washed with phosphate-buffered saline (PBS) to remove any unbound nanoparticles. Subsequently, they were incubated with DCFH-DA (25 µM) in DMEM at 37°C for 30 min. This incubation allowed the non-fluorescent probe to penetrate the cell membrane and be converted into the fluorescent compound DCF by intracellular ROS, enabling the assessment of oxidative stress levels.

#### 2.6.2 Measurement of MMP

MMP was assessed using the method established by Johnson et al. (Johnson et al., 1980). In this study, HCT-116 cells were treated with varying concentrations of PlS-ZnO NPs to investigate their effects on MMP. After treatment, the cells were stained with Rhodamine 123 dye and incubated for 15 min to facilitate effective dye uptake. Following incubation, the cells were washed with PBS to eliminate any excess dye and then fixed to preserve cellular architecture.

Fluorescence intensity was measured at an emission wavelength of 535 nm using a fluorescence plate reader. This measurement provided insights into MMP, with increased fluorescence intensity indicating intact mitochondrial function. The percentage of cells exhibiting pathological changes in MMP was determined by analyzing the fluorescence data, enabling the identification of cells experiencing depolarization or other adverse alterations due to the PlS-ZnO NPs treatment. This assessment contributed to a better understanding of the nanoparticles’ effects on mitochondrial dynamics within the cancer cells.

##### 2.6.2.1 Acridine orange/ethidium bromide (AO/EB) staining

The evaluation of apoptotic cell death was performed using a fluorescence microscopy technique based on the method described by Baskić et al. (2006). HCT-116 cells were initially seeded in a six-well tissue culture plate and incubated for 24 h. Following this, the cells were treated with varying concentrations of PlS-ZnO NPs (10 and 12.5 μg/mL) for an additional 24 h. After the treatment period, the cells were detached and washed with cold PBS to remove any unbound nanoparticles. The washed cells were then stained with a 1:1 mixture of acridine orange (AO) and ethidium bromide (EB) for 10 min at room temperature. This dual staining approach enables the differential visualization of live, apoptotic, and necrotic cells. The stained cells were subsequently examined under a fluorescence microscope at ×40 magnification. Apoptotic and necrotic cells were identified based on characteristic morphological features, such as chromatin condensation and membrane integrity. Cell counts for each category were recorded to quantify the extent of apoptosis and necrosis induced by the PlS-ZnO NP treatment, thereby providing insights into the mechanisms by which these nanoparticles induce cell death in HCT-116 cells.

##### 2.6.2.2 Propidium iodide (PI) staining

The evaluation of apoptotic cell death in HCT-116 cells treated with PlS-ZnO NPs, as well as in control groups, was performed using propidium iodide (PI) staining. Here, HCT-116 cells were cultured on each well of the 24-well plate for 24 h. After a day, different concentrations of (10 and 12.5 μg/mL) of PlS-ZnO NPs were added to the HCT-116 cells and cultured for further 24 h. Then the cells were stained using PI (5 µL) and stored carefully out of light for 20 min. Finally, PlS-ZnO NPs-induced apoptotic cell death was analyzed using a fluorescent microscope.

### 2.7 Determination of PlS-ZnO NPs antibacterial activity

PlS-ZnO NPs’ *in vitro* antibacterial activity was evaluated by agar well diffusion assay. The stock solution of ZnO NPs was prepared with DMSO (Dimethyl sulfoxide) to a concentration of 1 μg/μL. The selected pathogenic bacterial strains (*Bacillus megaterium NCIM 2087; Bacillus subtilis NCIM 2010; Vibrio cholerae ATCC 15748,* and *Proteus vulgaris NCIM 2027*) were maintained on a thin layer of solidified nutrient agar medium. A well of 10 mm diameter was cut on the agar surface. Different concentrations (75, 100, and 150 μg/mL) of PlS-ZnO NPs (50 µL/well) were added. Inhibition-forming zones were measured after 24 h. Amoxycillin was used as a control. The experiments were carried out in triplicates. The growth curve of bacteria was determined by exposing the selected bacterial species to PlS-ZnO NPs at 150 μg/mL concentration. Approximately 0.1 mL (10^8^ CFU/mL concentration) of bacterial suspension was inoculated with 100 mL of nutrient broth supplemented with PlS-ZnO NPs (150 μg/mL concentration). Bacterial cultures were incubated at 37°C for a 24-h period. Aliquots of the bacterial suspension were withdrawn at regular intervals, and their optical density was measured at 595 nm.

### 2.8 Statistical analysis

All experiments were performed in triplicate, with data expressed as mean ± standard error. Statistical analysis was conducted using one-way ANOVA, followed by Tukey’s *post hoc* test for multiple comparisons, utilizing GraphPad Prism version 8 (GraphPad Software, Inc., La Jolla, CA, United States).

## 3 Results

### 3.1 The phytochemicals identified in the stem of *P. lapathifolia*

Table 1 presents the phytochemicals identified in the stem of *P. lapathifolia* across various solvent extracts, including aqueous, methanol, and chloroform. The aqueous extract revealed the presence of carbohydrates, flavonoids, saponins, steroids, tannins, and terpenoids. In contrast, the methanol extract contained carbohydrates, proteins, saponins, flavonoids, alkaloids, glycosides, and tannins, but lacked steroids and terpenoids. The chloroform extract showed proteins, saponins, flavonoids, steroids, and tannins. Overall, the preliminary analysis indicates that the stem of *P. lapathifolia* is rich in phytochemicals, which likely contribute to its pharmacological properties.

**TABLE 1:** Phytochemical screening of *P. lapathifolia* stem using different solvent extracts.

Phytochemicals	Aqueous	Methanol	Chloroform
Carbohydrates	+	+	-
Proteins	-	+	+
Saponins	+	+	+
Alkaloids	-	+	-
Flavonoids	+	+	+
Glycosides	-	+	-
Steroids	+	-	+
Tannins	+	+	+
Terpenoids	+	-	-

### 3.2 Biosynthesis and characterization of PIS-ZnO nanoparticles

**FIGURE 1:**
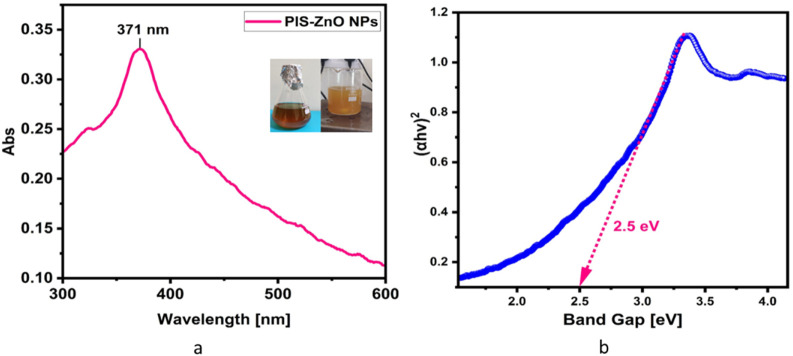
**(a)** UV-visible spectrum of *P. lapathifolia*-synthesized ZnO nanoparticles alongside a visual representation of the reaction mixture. The observed color change from dark brown to pale yellow in the spectrum confirms the successful formation of ZnO phytocomplex. The precipitate after calcination turned into a white powder indicating the formation of Zinc oxide nanoparticles. **(b)** Tauc plot of synthesized ZnO NPs using *P. lapathifolia* stem extract.

The synthesis of PlS-ZnO nanoparticles (NPs) was indicated by the appearance of a pale-yellow solution, which signified the formation of a zinc-phytocomplex. After calcination at 600°C for 2 hours, a white powder of ZnO NPs was obtained. The UV-visible spectrum displayed a significant peak at 371 nm, confirming the successful formation of PlS-ZnO NPs (Figure 1a). The energy gap of these nanoparticles was measured to be 2.5 eV, as derived from the Tauc plot, which involved plotting (*α*ℎ*ν*)2 against ℎ*ν* (Figure 1b).

**FIGURE 2:**
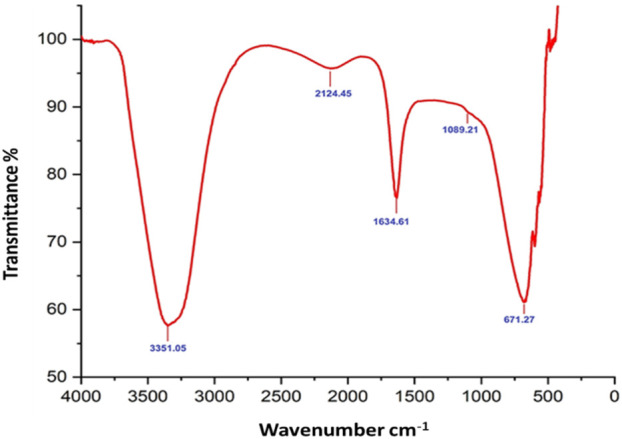
FTIR spectrum of *P. lapathifolia*-synthesized ZnO nanoparticles.

**FIGURE 3:**
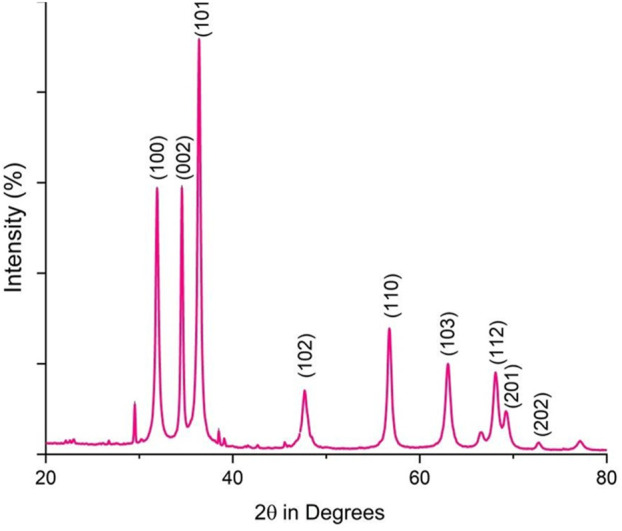
XRD pattern of *P. lapathifolia*-synthesized ZnO nanoparticles.

Fourier-transform infrared (FTIR) analysis revealed several peaks at wavenumbers of 3351.05, 2124.45, 1634.61, 1089.21, and 671.27 cm^−1^ (Figure 2), indicating the presence of various functional groups. X-ray diffraction (XRD) analysis confirmed the successful synthesis of the zinc oxide nanoparticles from the *P. lapathifolia* stem extract. The diffraction pattern (Figure 3) aligned with the standard powder diffraction card JCPDS No: 80.0075, confirming a crystalline hexagonal wurtzite structure typical of ZnO. Using the Scherrer equation, the average crystallite size was calculated to be 14.50 nm. High-resolution XRD revealed distinct peaks at 2θ values of 31.77°, 34.42°, 36.25°, 47.55°, 56.60°, 62.86°, 66.37°, 67.96°, and 69.09°, corresponding to various crystallographic planes of the hexagonal ZnO structure. The sharpness and intensity of these peaks indicated that the synthesized nanoparticles were highly crystalline and pure.

**FIGURE 4:**
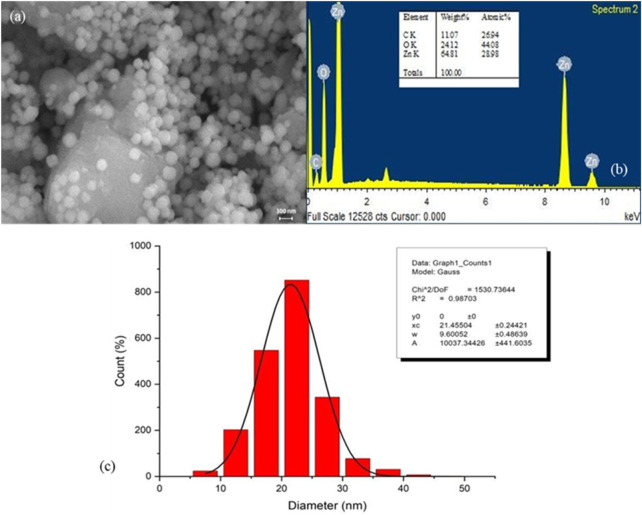
Structure, elemental composition, and size of *P. lapathifolia*-synthesized ZnO nanoparticles: **(a)** FESEM micrograph, **(b)** EDX spectrum, and **(c)** Particle size distribution.

Field emission scanning electron microscopy (FESEM) was employed to examine the surface morphology of the synthesized PlS-ZnO NPs. The FESEM images (Figure 4a) displayed a combination of polygonal and hexagonal nanostructures, suggesting effective stabilization and capping by the phytochemicals from the plant extract. Larger particles were often found associated with smaller ones, indicating successful encapsulation by bioactive compounds. Energy-dispersive X-ray (EDX) spectroscopy further analyzed the elemental composition of the nanoparticles (Figure 4b). The EDX spectrum confirmed the presence of zinc (64.81%), oxygen (24.1%), and carbon (11.0%), reinforcing the purity of the synthesized NPs. The absence of extraneous peaks in the EDX data further emphasized the high quality of the green-synthesized PlS-ZnO NPs.

The size distribution of the nanoparticles was assessed using ImageJ software, yielding an average particle size of 21.46 ± 0.24 nm (Figure 4c). This narrow size distribution is advantageous for biomedical and nanotechnology applications, as it ensures consistent and predictable performance. In summary, the findings from FESEM, EDX, and particle size analysis collectively affirm the successful green synthesis of pure, well-defined, and size-controlled zinc oxide nanoparticles using the *P. lapathifolia* stem extract. The unique morphological, compositional, and dimensional characteristics of the PlS-ZnO NPs indicate their potential for further exploration in biomedicine and advanced materials science.

### 3.3 Evaluation of the anticancer potential of phyto-synthesized ZnO nanoparticles

#### 3.3.1 Cytotoxic efficacy of PlS-ZnO NPs against CRC cells

**FIGURE 5:**
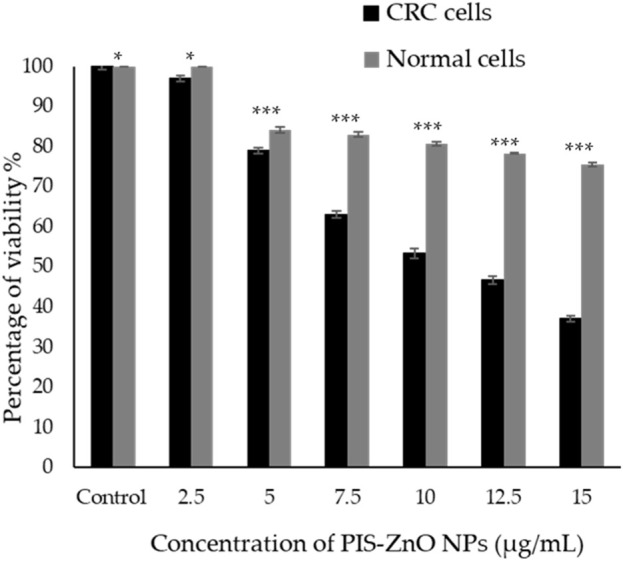
The cytotoxic effects of the phytogenic zinc oxide nanoparticles (PlS-ZnO NPs) were assessed against the human colorectal carcinoma (HCT-116) cell line and normal colon (CCD 841 CoN) cells using the MTT assay. The data presented represents the mean ± standard deviation (SD) of three independent experimental replicates. Statistical significance was determined through Tukey’s multiple comparisons test, where * indicates a significant difference at p = 0.018, and *** denotes high statistical significance at p < 0.001.

The antiproliferative effects of phytogenic zinc oxide nanoparticles (PlS-ZnO NPs), synthesized from *P. lapathifolia* stem extract, were investigated against human colorectal carcinoma (HCT-116) cells using the MTT assay (Figure 5). The results showed that PlS-ZnO NPs exerted a concentration-dependent cytotoxic effect on CRC cells. As the concentration of the nanoparticles increased from 2.5 to 15 μg/mL, a notable decrease in cell viability was observed. At the lowest concentration of 2.5 μg/mL, HCT-116 cell viability remained high at 97%. However, when the concentration reached 15 μg/mL, cell viability plummeted to 37%. The half-maximal inhibitory concentration (IC50) for PlS-ZnO NPs against HCT-116 cells was determined to be 11.31 μg/mL.

In comparison, normal human colon cells (CCD 841 CoN) showed a higher viability of 75.45% ± 0.51% even at the maximum concentration of 15 μg/mL of PlS-ZnO NPs. Statistical analysis using Tukey’s multiple comparisons test revealed significant differences in cytotoxic effects between the CRC and normal cells (p = 0.018 and p < 0.001).

These results suggest that the green-synthesized PlS-ZnO NPs selectively target HCT-116 cells, demonstrating strong antiproliferative properties while exhibiting lower toxicity toward normal colon cells. This selectivity is particularly advantageous for cancer therapy, as it minimizes potential off-target effects and enhances the therapeutic efficacy of the treatment.

#### 3.3.2 Cytomorphological alterations in colorectal cancer cells induced by PlS-ZnO NPs

**FIGURE 6:**
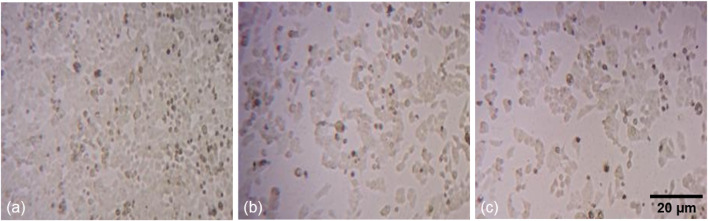
Fluorescence microscopic images of morphological alterations in HCT-116 treated with PlS-ZnO NPs: **(a)** control, **(b)** PlS-ZnO NPs (10 μg/mL), and **(c)** PlS-ZnO NPs (12.5 μg/mL).

To assess the cellular responses of human colorectal carcinoma (HCT-116) cells exposed to two concentrations (10 and 12.5 μg/mL) of phyto-synthesized PlS-ZnO NPs, detailed cytomorphological analyses were performed. Observations under phase-contrast microscopy revealed striking morphological changes in the treated HCT-116 cells when compared to the untreated control group (Figures 6a–c).

The cells treated with PlS-ZnO NPs showed a notable loss of adhesion to the culture surface, which could indicate the induction of anoikis, a form of programmed cell death triggered when cells detach from the extracellular matrix. Additionally, the treated cells exhibited shrunken and distorted shapes, reflecting significant cellular damage. The appearance of large, distinct cellular blebs further suggested that necrotic cell death was occurring in these HCT-116 cells.

These collective cytomorphological changes—loss of adherence, cell deformation, and necrotic blebbing—strongly indicate that treatment with PlS-ZnO NPs activated both apoptotic and necrotic pathways in HCT-116 cells, which were absent in the untreated control cells.

#### 3.3.3 Modulation of intracellular ROS and mitochondrial membrane potential (MMP) in colorectal cancer cells by *P. lapathifolia*-synthesized ZnO nanoparticles

**FIGURE 7:**
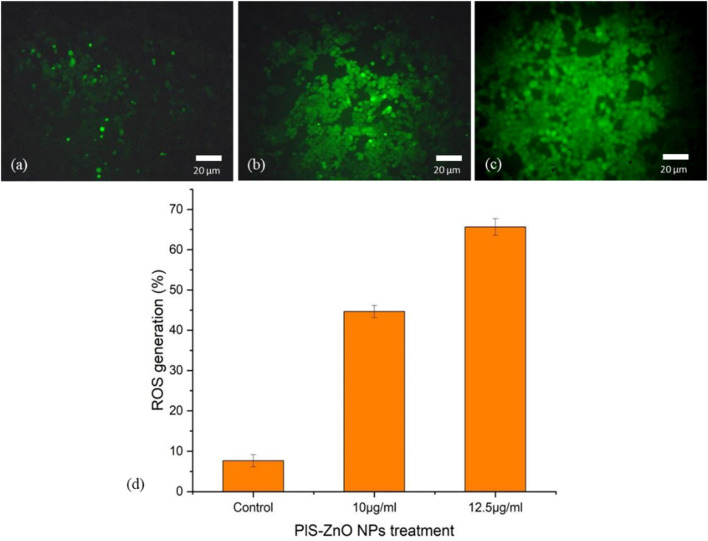
Fluorescence microscopic images of ROS generation in HCT-116 cells treated with PlS-ZnO NPs: **(a)** control, **(b)** PlS-ZnO NPs (10 μg/mL, **(c)** PlS-ZnO NPs (12.5 μg/mL). and **(d)** Histogram represents the quantification of apoptotic cells in percentage.

The potential of phyto-synthesized PlS-ZnO nanoparticles (NPs) to induce oxidative stress and impair mitochondrial function in human colorectal carcinoma (HCT-116) cells was assessed through fluorescence-based assays. Using the DCFH-DA assay, researchers quantified the levels of intracellular ROS. The findings indicate a significant, concentration-dependent increase in ROS levels following treatment with PlS-ZnO nanoparticles (NPs). Specifically, ROS levels were observed to increase to 45% and 65.67% in cells treated with 10 μg/mL and 12.5 μg/mL of PlS-ZnO NPs, respectively, compared to only 8% in the untreated control group (Figures 7a–d).

**FIGURE 8:**
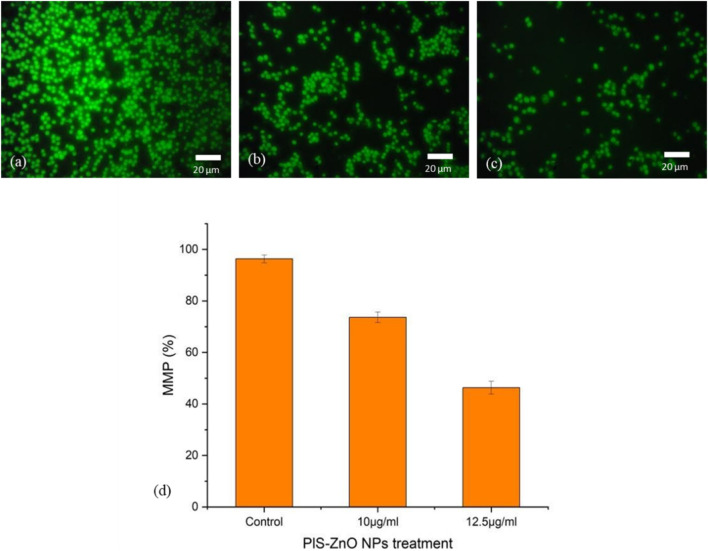
Fluorescence microscopic images of alterations in MMP of HCT-116 treated with PlS- ZnO NPs analyzed using rhodamine 123 stain: **(a)** control, **(b)** PlS-ZnO NPs (10 μg/mL), **(c)** PlS-ZnO NPs (12.5 μg/mL) and **(d)** Histogram represents the quantification of MMP in percentage.

Additionally, the impact of PlS-ZnO NPs on the mitochondrial membrane potential (MMP) of HCT-116 cells was assessed using the lipophilic cationic dye Rhodamine-123. Treatment with 10 μg/mL and 12.5 μg/mL of PlS-ZnO NPs resulted in a significant decrease in MMP, falling from 98% in the control group to 77.5% and 49%, respectively (Figures 8a–d).

Overall, these results indicate that PlS-ZnO NPs effectively induce oxidative stress and impair mitochondrial function in HCT-116 colorectal cancer cells in a concentration-dependent manner, which may contribute to the observed cytotoxic effects.

#### 3.3.4 Induction of apoptosis and necrosis in colorectal cancer cells by *P. lapathifolia*-synthesized ZnO nanoparticles

The mechanism of cell death triggered by phyto-synthesized PlS-ZnO nanoparticles (NPs) in human colorectal carcinoma (HCT-116) cells was examined using a dual staining technique with fluorescent dyes, Acridine Orange (AO) and Ethidium Bromide (EtBr). Microscopic analysis of the stained cells revealed distinct fluorescence patterns associated with different cell death pathways. Cells with intact nuclei showed bright green fluorescence from AO staining, indicating they were viable. Conversely, cells exhibiting nuclear fragmentation, a key feature of apoptosis, displayed orange fluorescence due to EtBr staining. Additionally, necrotic cells were marked by an orange-to-red fluorescence, signaling a loss of plasma membrane integrity.

**FIGURE 9:**
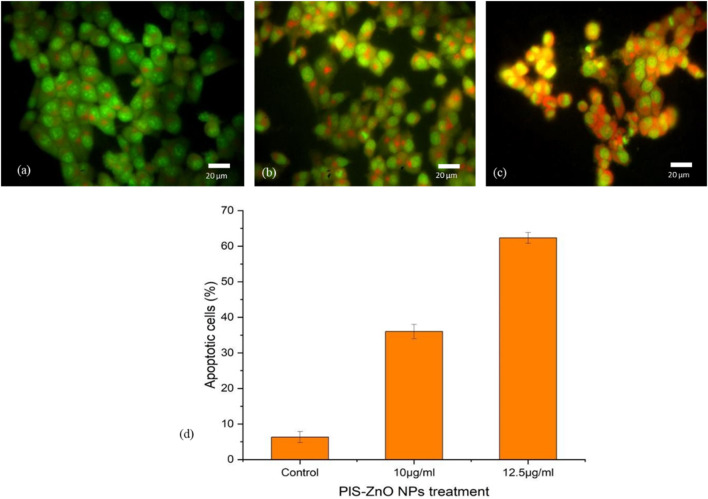
Morphological assessment of apoptosis and necrosis in HCT-116 cells treated with *P. lapathifolia*-synthesized ZnO nanoparticles. Bright-field microscopy images were captured following AO/EtBr staining. **(a)** Control, **(b)** PlS-ZnO NPs (10 μg/mL) and **(c)** PlS-ZnO NPs (12.5 μg/mL). **(d)** Quantitative analysis of apoptotic cells as a percentage of the total cell population.

Importantly, HCT-116 cells treated with a higher concentration of 12.5 μg/mL PlS-ZnO NPs—above the calculated IC50—showed a significant increase in cell death, reaching 64%. This outcome was attributed to the induction of both apoptosis and necrosis (Figures 9a–d). These results illustrate that PlS-ZnO NPs can effectively initiate programmed cell death (apoptosis) as well as necrotic cell death in HCT-116 cells in a concentration-dependent manner, underscoring their potent cytotoxic properties.

#### 3.3.5 Propidium iodide (PI) staining

To further investigate the induction of apoptosis in HCT-116 cells by phyto-synthesized PlS-ZnO nanoparticles (NPs), propidium iodide (PI) staining was employed, which is a well-known marker for detecting apoptotic cells. Fluorescence microscopy revealed that HCT-116 cells treated with PlS-ZnO NPs exhibited intense red fluorescence, indicative of fragmented nuclei and the activation of apoptosis. Notably, the intensity of this red fluorescence increased in a concentration-dependent manner, suggesting that higher doses led to a greater apoptotic response.

**FIGURE 10:**
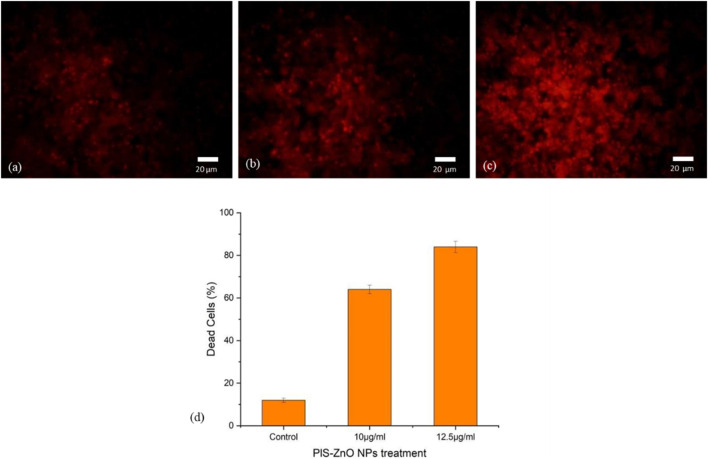
Fluorescence microscopic images of HCT-116 treated with PlS-ZnO NPs analyzed using PI staining: **(a)** control, **(b)** PlS-ZnO NPs (10 μg/mL), **(c)** PlS-ZnO NPs (12.5 μg/mL), and **(d)** Histogram represents the quantification of apoptotic cells in percentage.

In contrast, untreated control HCT-116 cells displayed a much dimmer red fluorescence, indicating a lower level of apoptosis compared to their treated counterparts. Quantitative analysis of the PI staining data showed that after 24 h of exposure, 83% of HCT-116 cells treated with the higher concentration of 12.5 μg/mL PlS-ZnO NPs had undergone apoptosis (Figures 10a–d). These results strongly support the conclusion that PlS-ZnO NPs effectively induce apoptotic cell death in human colorectal cancer cells in a concentration-dependent manner, highlighting their significant cytotoxic potential against this aggressive cancer type.

### 3.4 Potent antimicrobial efficacy of phyto-synthesized PlS-ZnO nanoparticles

**FIGURE 11:**
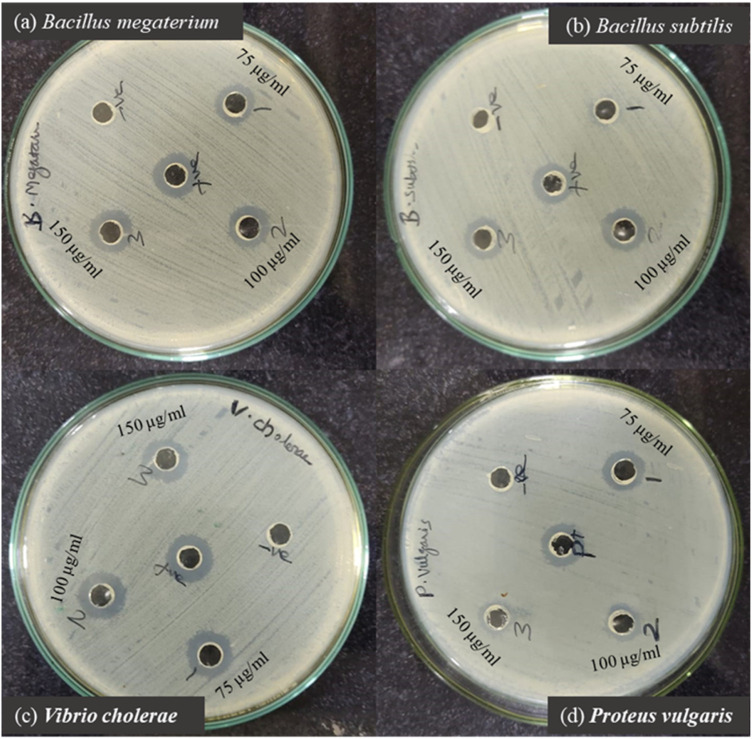
Zone of inhibition produced by PIS-ZnO NPs against selected bacterial pathogens. **(a)** *Bacillus megaterium*, **(b)** *Bacillus subtilis*, **(c)** *Vibrio cholerae*, and **(d)** *Proteus vulgaris*.

The antibacterial properties of phyto-synthesized PlS-ZnO nanoparticles (NPs) were thoroughly assessed using a robust agar well diffusion assay. Various concentrations of PlS-ZnO NPs (75, 100, and 150 μg/mL) were tested against a range of clinically relevant bacterial strains, including *B*. *megaterium*, *P*. *vulgaris*, *V*. *cholerae*, and *B*. *subtilis* (Figures 11a–d). The results showed significant growth inhibition zones around the wells containing the PlS-ZnO NPs for all tested bacteria. Notably, *B. subtilis* exhibited the greatest susceptibility, with an inhibition zone measuring 16.3 ± 0.58 mm. In contrast, *B. megaterium* showed the least susceptibility, with an inhibition zone of 15 ± 1 mm. Both *V. cholerae* and *P. vulgaris* exhibited intermediate susceptibility, with inhibition zones of 16 ± 0.4 mm and 15 ± 2 mm, respectively (Table 2).

**TABLE 2:** Antibacterial activity of PlS-ZnO nanoparticles against selected bacterial strains.

	Inhibition zone (mm)			
	*B. subtilis*	*B. megaterium*	*V. cholerae*	*P. vulgaris*
75 μg/mL	13.5 ± 0.25	13.8 ± 0.3	14.3 ± 0.15	14.2 ± 0.15
100 μg/mL	15.7 ± 0.1	14 ± 0.2	15 ± 0.3	14.8 ± 0.1
150 μg/mL	16.3 ± 0.58	15 ± 1	16 ± 0.4	15 ± 2
Amoxycillin (20 mg/μL)	18 ± 0.1	15.7 ± 0.1	18.5 ± 0.15	16.5 ± 0.3

**FIGURE 12:**
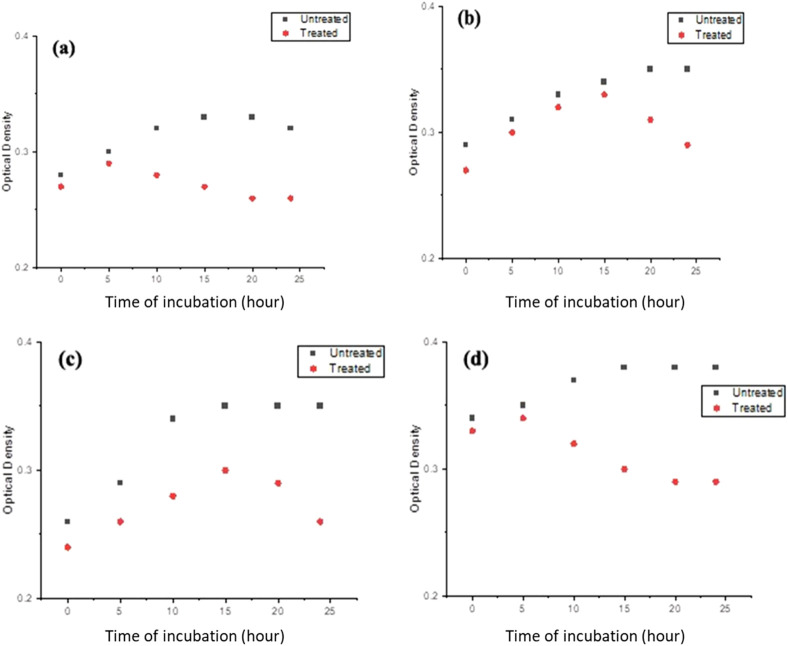
The growth kinetics of **(a)** *Bacillus megaterium*, **(b)** *Proteus vulgaris*, **(c)** *Vibrio cholerae*, and **(d)** *Bacillus subtilis* were evaluated in the presence and absence of PlS-ZnO NPs at a concentration of 150 μg/mL.

Further investigation of the bacterial growth kinetics revealed a gradual, concentration-dependent reduction in the growth of these bacterial species when exposed to PlS-ZnO NPs at 150 μg/mL (Figures 12a–d). Compared to the control, the bacterial cultures treated with PlS-ZnO NPs exhibited notable changes in both the lag and exponential growth phases, as indicated by significant reductions in absorbance measurements.

These findings strongly suggest that phyto-synthesized PlS-ZnO nanoparticles possess potent broad-spectrum antimicrobial activity, effectively inhibiting the growth of diverse bacterial pathogens. This impressive antibacterial efficacy highlights the potential of these nanoparticles as promising antimicrobial agents for various biomedical and clinical applications.

## 4 Discussion

The phytochemical screening of the stem extract was conducted using both high and low polar solvents, revealing the presence of carbohydrates, proteins, flavonoids, saponins, alkaloids, glycosides, steroids, tannins, and terpenoids. The phytochemical profile obtained from the methanol extract aligns with the findings of Shaira et al. (Shaira et al., 2023). Additionally, the composition of the aerial parts of the plant, as investigated by Khan et al. (2024), corroborates our results. However, the aqueous extract of the aerial parts did not contain flavonoids, which differs from our findings, while the chloroform extract showed consistency with our data. The observed antibacterial and anticancer activities of the plant may be attributed to the presence of flavonoids and glycosides (Ahmed et al., 2016).

Nanomaterials, particularly ZnO NPs synthesized through green methods, have garnered significant interest in various fields, including medicine, due to their enhanced surface-to-volume ratio. Compared to other metal oxide nanoparticles, ZnO NPs are cost-effective and exhibit lower toxicity. Their biodegradability and biocompatibility further enhance their appeal for pharmaceutical applications (Anjum et al., 2021).

In this study, the phytochemicals in *P. lapathifolia* stems were utilized for the synthesis of ZnO NPs, using zinc nitrate as the metal precursor. The literature supports the pharmacological potential of *P. lapathifolia* (Abd-ElGawad et al., 2021; Seimandi et al., 2021). We employed a simple sol-gel technique for synthesizing PlS-ZnO NPs, relying solely on the aqueous stem extract without hazardous chemicals. Phytochemicals such as aldehydes, alkaloids, flavonoids, terpenoids, saponins, glycosides, tannins, and sterols play crucial roles in reducing the metal precursor to nanoparticles, capping, and stabilizing the synthesized NPs (Patel et al., 2022). The dissolution of zinc nitrate in water leads to the dissociation of zinc (Zn^2+^) and nitrate (NO_3_^−^) ions, with the former interacting with phytochemicals in the extract, facilitating the reduction of zinc ions to zinc hydroxide. Subsequent calcination converts zinc hydroxide into zinc oxide nanoparticles. Preliminary analyses indicate that the aqueous stem extract is rich in phenolic compounds, which are likely essential for the reduction process and subsequent synthesis of zinc oxide nanoparticles (Barzinjy and Azeez, 2020).

The sol-gel method using aqueous stem extract for ZnO NP synthesis has been well-documented (Al-darwesh et al., 2024; Ismail et al., 2019; MuthuKathija et al., 2023). Variations in particle size, morphology, and biological activity of phyto-mediated NPs are primarily linked to differences in phytochemical composition and concentration (Neamah et al., 2023). In our study, comprehensive characterization techniques, including XRD, UV-visible spectroscopy, and scanning electron microscopy (SEM), confirmed the synthesis, size, crystallinity, purity, and morphology of the ZnO NPs. The UV spectrum exhibited peaks at 315 and 371 nm, confirming the formation of ZnO NPs. Similarly, Limonia acidissima leaf-mediated ZnO NPs displayed an absorbance peak at 374 nm (Al Rahbi et al., 2024). The FTIR spectrum indicated the presence of various functional groups, including alcohols, amines, alkanes, carboxylic acids, and metal oxides, further supporting the characterization of ZnO NPs. A broad absorption peak at 3351 cm^−1^ suggests a high concentration of alcohols, reflecting OH^−^ stretches and/or N-H stretches from amines (Mamand et al., 2025). The less intense peak at 2,124 cm^−1^ corresponds to C-H stretching vibrations typical of alkanes. Additionally, the sharp peak at 1,634 cm^−1^ is attributed to C=O stretching vibrations in carboxylic acids (Al-Nemrawi et al., 2022). The peak at 1,089 cm^−1^ is associated with C-O stretching vibrations of alcohol (Abomuti et al., 2021), while the peak at 671 cm^−1^ is due to the metal oxide stretching vibrations of ZnO NPs (Najoom et al., 2021). The XRD pattern confirmed that the PlS-ZnO NPs are highly crystalline and possess a hexagonal wurtzite structure, consistent with JCPDS card no. 80–0075 (Sundaram et al., 2020). The average particle sizes calculated using the Scherrer equation were 18.03 nm and 14.50 nm. The SEM analysis of PlS-ZnO NPs revealed particles with polygonal and hexagonal structures that are agglomerated, with a particle size of 21.45 nm as determined by ImageJ, and a size distribution ranging from 5 to 45 nm (Ahlam et al., 2021). This indicates a relatively uniform dispersion of particles, which is important for their potential applications.

This pioneering investigation highlights the significant antiproliferative effects of ZnO nanoparticles synthesized from *P. lapathifolia* stem extract on HCT-116 colorectal cancer cells. Our analyses demonstrate that PlS-ZnO NPs exert cytotoxic effects by inducing elevated levels of intracellular ROS, compromising membrane integrity, and triggering apoptosis. The cytotoxicity of PlS-ZnO NPs was evaluated using the MTT assay, which revealed a statistically significant increase in cytotoxicity, with the half-maximal inhibitory concentration (IC_50_) calculated at 11.31 μg/mL. We further examined the effects of concentrations around the IC_50_, specifically 10 μg/mL and 12.5 μg/mL, on HCT-116 cells. Microscopic analysis indicated the activation of both apoptotic and necrotic pathways, consistent with previous studies on phytochemical-mediated ZnO NPs in various cancer cell lines including MCF-7 (Farasat et al., 2020), HCT-116, K562 and MDA- MB-468 cells (Gupta et al., 2023). The enhanced cytotoxicity and ROS generation observed after 24 h of exposure suggest that oxidative stress plays a crucial role in the observed cellular damage.

Molecular analysis of oxidative stress-related ROS generation and loss of MMP provided insights into the overlapping biochemical pathways influenced by PlS-ZnO NP treatment. The small particle size and large surface area of ZnO NPs are associated with increased ROS production and oxidative stress. ZnO NPs can generate both radical and non-radical ROS, activating signaling pathways that influence apoptosis and cell division (Dorier et al., 2017; Liu et al., 2020). The cytotoxicity of ZnO NPs is largely attributed to excessive ROS generation, which acts as redox messengers regulating cellular signaling pathways (Vandebriel and De Jong, 2012; Zhang et al., 2017). Additionally, the hydrodynamic size of ZnO NPs is significant in promoting ROS production within mitochondria (Liu et al., 2024). In this study, we assessed the ability of PlS-ZnO NPs to generate intracellular ROS using the DCFH-DA assay, and the results were consistent with previous report on biogenic NPs (Lakshmipriya et al., 2018).

The release of transition metal ions from NPs has been linked to their cytotoxicity (Naser et al., 2023) and ability to increase intracellular ROS in cancer cells through Fenton reactions (Min et al., 2023). However, studies indicate that the released Zn^2+^ ions alone are insufficient to cause significant cytotoxicity. The semiconductor properties and surface characteristics of ZnO NPs enable spontaneous ROS generation. The observed increase in ROS production and oxidative stress in cancer cells exposed to ZnO NPs can be attributed to the presence of electron-hole pairs caused by size reduction, leading to the generation of hydroxyl and superoxide radicals that can penetrate cell membranes and damage DNA (Khan et al., 2021). ROS have the potential to indirectly impact DNA integrity. Growing evidence indicates that ZnO nanoparticles (NPs) can trigger cell death through apoptotic pathways, as evidenced by observations of nuclear condensation and fragmentation. The selective toxicity of PlS-ZnO NPs toward HCT-116 colorectal cancer cells, as compared to normal colon epithelial cells (CCD 841 CoN), can be attributed to several well-established biological factors. Cancer cells typically exhibit higher basal levels of oxidative stress and are more susceptible to further increases in ROS. ZnO nanoparticles are known to induce ROS generation, which selectively pushes cancer cells beyond their oxidative stress threshold, leading to apoptosis, while normal cells can often mitigate moderate ROS through more effective antioxidant responses (Akhtar et al., 2012).

Additionally, cancer cells demonstrate enhanced nanoparticle uptake due to their increased metabolic activity and altered membrane permeability, resulting in greater intracellular accumulation and cytotoxic effects. Moreover, the phytochemicals present in the *P. lapathifolia* stem extract may contribute synergistically to mitochondrial dysfunction and apoptosis in cancer cells, further enhancing selectivity (Adjei et al., 2014). These findings highlight the role of oxidative stress in mediating cellular damage and apoptosis in response to ZnO NP exposure (George et al., 2022).

We also observed alterations in MMP in HCT-116 cells treated with PlS-ZnO NPs. Mitochondria are critical for ATP synthesis, cellular signaling, and growth regulation. The reduction in MMP, as indicated by the Rhodamine 123 assay, suggests that oxidative phosphorylation in the mitochondrial membrane is a key mechanism underlying the cytotoxic effects of ZnO NPs in HCT-116 cells (Canaparo et al., 2020). Overall, these findings indicate that ZnO NPs induce a ROS-sensitized mitochondrial pathway as a primary mechanism of cell death in HCT-116 cells (Berehu and Patnaik, 2024).

Extensive research demonstrates the strong antibacterial activity of green-synthesized PlS-ZnO nanoparticles (NPs), attributed to multiple mechanisms (Gangadoo et al., 2022; Krishnamoorthy et al., 2022; Mendes et al., 2022; Shanmugam et al., 2023). One proposed mechanism is the interaction of ZnO NPs with bacterial membrane lipids, which disrupts cellular integrity, resulting in compromised membrane function and eventual cell death (Gangadoo et al., 2022). Additionally, it is suggested that ZnO NPs can penetrate bacterial membranes, where they generate free radicals that inflict significant damage to DNA, membranes, and proteins, ultimately leading to the inhibition of bacterial growth (Mendes et al., 2022).

In our study, PlS-ZnO NPs exhibited significant bactericidal activity against both Gram-positive and Gram-negative bacterial species. Although the inhibition zones varied slightly between the two groups, the results demonstrate the broad-spectrum antibacterial efficacy of the PlS-ZnO NPs. Specifically, *B. subtilis* (Gram-positive) and *V. cholerae* (Gram-negative) showed inhibition zones of 16.3 ± 0.58 mm and 16 ± 0.58 mm, respectively, while *B. megaterium* (Gram-positive) and *P. vulgaris* (Gram-negative) displayed zones of 15 ± 1 mm and 15.3 ± 2 mm, respectively. In the absence of PlS-ZnO NPs, the bacterial growth curves indicated significant increases in absorbance, whereas those exposed to PlS-ZnO NPs showed marked reductions in absorbance, likely due to stress induced by the ZnO NPs (Krishnamoorthy et al., 2022). These findings corroborate the potent toxic effects of PlS-ZnO NPs on the selected bacterial species, consistent with previous studies reporting impaired growth kinetics in bacteria treated with ZnO NPs (Shanmugam et al., 2023). Our results suggest that ZnO NPs have the potential to combat bacterial infections and help mitigate the growing global threat of antimicrobial resistance (Khalifa et al., 2021; Khalifa et al., 2022; Al-Hakkani et al., 2023; Khalifa et al., 2025).

## 5 Conclusion

This study successfully demonstrates the eco-friendly synthesis of zinc oxide nanoparticles (PlS-ZnO NPs) using an aqueous extract from *P. lapathifolia* stems. The synthesized nanoparticles exhibit a unique polygonal and hexagonal morphology, with an average size of 21.45 nm, and show impressive biocompatibility with normal cells alongside potent selective cytotoxic effects against the HCT-116 colorectal cancer cell line, achieving an IC_50_ value of 11.31 μg/mL. Mechanistic investigations reveal that PlS-ZnO NPs induce dual modes of cell death—apoptosis and necrosis—mediated by increased levels of ROS and disruption of MMP. Furthermore, these nanoparticles demonstrate significant antibacterial activity against several pathogenic bacteria, including *B. subtilis*, *B. megaterium*, *V. cholerae*, and *P. vulgaris*, surpassing the efficacy of the positive control, amoxicillin. This highlights the multifunctional potential of green-synthesized PlS-ZnO NPs as promising therapeutic agents for combating both colorectal cancer and bacterial infections. The inherent limitations of conventional synthetic anticancer drugs characterized by non-specific distribution, adverse side effects, and suboptimal efficacy have impeded advancements in cancer treatment. The unique surface chemistry imparted by phytochemicals enhances the interaction of these nanoparticles with biological systems, facilitating targeted therapeutic effects. The environmentally friendly and cost-effective synthesis method not only enhances the therapeutic prospects of PlS-ZnO NPs but also underscores the translational potential of utilizing natural resources in nanomedicine. Future research should concentrate on optimizing nanoparticle formulations, conducting *in vivo* studies to further evaluate efficacy and safety, and addressing potential toxicity concerns to fully leverage their therapeutic potential in the complex landscape of cancer and infectious disease management.

## Data Availability

The original contributions presented in the study are included in the article/supplementary material, further inquiries can be directed to the corresponding author.
